# Supplementary material for: Unveiling the Bioactive Potential of the Invasive Jellyfish Phyllorhiza punctata Through Integrative Transcriptomic and Proteomic Analyses
Source: Biomolecules. 2025 Aug 4;15(8):1121. doi: 10.3390/biom15081121 (PMC12383608; doi:10.3390/biom15081121)
Supplement: Supplementary file 1 [file biomolecules-15-01121-s001.zip › Suplementary Material/FileS3/PEPFOLD4/XP_065069482_4468/clusters.html]

 PEP-FOLD clusters


Download archive of all models  here   

|  |  |  |
| --- | --- | --- |
| # | file | sOPEP |
| # cluster 1 - Size: 1 |  |  |
|  | XP\_065069482\_4468-model1 | -10.0542 |
| # cluster 2 - Size: 2 |  |  |
|  | XP\_065069482\_4468-model2 | -8.10604 |
|  | XP\_065069482\_4468-model2.1 | -6.06672 |
| # cluster 3 - Size: 8 |  |  |
|  | XP\_065069482\_4468-model3 | -7.92529 |
|  | XP\_065069482\_4468-model3.1 | -7.67217 |
|  | XP\_065069482\_4468-model3.2 | -6.76913 |
|  | XP\_065069482\_4468-model3.3 | -6.36702 |
|  | XP\_065069482\_4468-model3.4 | -6.30265 |
|  | XP\_065069482\_4468-model3.5 | -6.08241 |
|  | XP\_065069482\_4468-model3.6 | -5.66991 |
|  | XP\_065069482\_4468-model3.7 | -5.60564 |
| # cluster 4 - Size: 7 |  |  |
|  | XP\_065069482\_4468-model4 | -7.38711 |
|  | XP\_065069482\_4468-model4.1 | -7.16902 |
|  | XP\_065069482\_4468-model4.2 | -6.81591 |
|  | XP\_065069482\_4468-model4.3 | -6.8081 |
|  | XP\_065069482\_4468-model4.4 | -6.78236 |
|  | XP\_065069482\_4468-model4.5 | -6.14277 |
|  | XP\_065069482\_4468-model4.6 | -5.10986 |
| # cluster 5 - Size: 28 |  |  |
|  | XP\_065069482\_4468-model5 | -7.34147 |
|  | XP\_065069482\_4468-model5.1 | -7.09504 |
|  | XP\_065069482\_4468-model5.2 | -6.79621 |
|  | XP\_065069482\_4468-model5.3 | -6.6238 |
|  | XP\_065069482\_4468-model5.4 | -6.50379 |
|  | XP\_065069482\_4468-model5.5 | -6.50379 |
|  | XP\_065069482\_4468-model5.6 | -6.43712 |
|  | XP\_065069482\_4468-model5.7 | -6.3463 |
|  | XP\_065069482\_4468-model5.8 | -6.31656 |
|  | XP\_065069482\_4468-model5.9 | -6.31428 |
|  | XP\_065069482\_4468-model5.10 | -6.28665 |
|  | XP\_065069482\_4468-model5.11 | -6.17084 |
|  | XP\_065069482\_4468-model5.12 | -6.09776 |
|  | XP\_065069482\_4468-model5.13 | -6.0683 |
|  | XP\_065069482\_4468-model5.14 | -6.04665 |
|  | XP\_065069482\_4468-model5.15 | -5.87783 |
|  | XP\_065069482\_4468-model5.16 | -5.86194 |
|  | XP\_065069482\_4468-model5.17 | -5.75145 |
|  | XP\_065069482\_4468-model5.18 | -5.74038 |
|  | XP\_065069482\_4468-model5.19 | -5.69048 |
|  | XP\_065069482\_4468-model5.20 | -5.59945 |
|  | XP\_065069482\_4468-model5.21 | -5.52964 |
|  | XP\_065069482\_4468-model5.22 | -5.47334 |
|  | XP\_065069482\_4468-model5.23 | -5.30603 |
|  | XP\_065069482\_4468-model5.24 | -5.24468 |
|  | XP\_065069482\_4468-model5.25 | -5.01869 |
|  | XP\_065069482\_4468-model5.26 | -4.83839 |
|  | XP\_065069482\_4468-model5.27 | -4.58318 |
| # cluster 6 - Size: 1 |  |  |
|  | XP\_065069482\_4468-model6 | -7.08444 |
| # cluster 7 - Size: 2 |  |  |
|  | XP\_065069482\_4468-model7 | -6.85159 |
|  | XP\_065069482\_4468-model7.1 | -5.36317 |
| # cluster 8 - Size: 4 |  |  |
|  | XP\_065069482\_4468-model8 | -6.78176 |
|  | XP\_065069482\_4468-model8.1 | -6.30214 |
|  | XP\_065069482\_4468-model8.2 | -5.95061 |
|  | XP\_065069482\_4468-model8.3 | -5.90733 |
| # cluster 9 - Size: 6 |  |  |
|  | XP\_065069482\_4468-model9 | -6.56061 |
|  | XP\_065069482\_4468-model9.1 | -6.54975 |
|  | XP\_065069482\_4468-model9.2 | -6.27371 |
|  | XP\_065069482\_4468-model9.3 | -6.1843 |
|  | XP\_065069482\_4468-model9.4 | -5.50082 |
|  | XP\_065069482\_4468-model9.5 | -5.42283 |
| # cluster 10 - Size: 17 |  |  |
|  | XP\_065069482\_4468-model10 | -6.53179 |
|  | XP\_065069482\_4468-model10.1 | -6.43747 |
|  | XP\_065069482\_4468-model10.2 | -6.29375 |
|  | XP\_065069482\_4468-model10.3 | -6.20743 |
|  | XP\_065069482\_4468-model10.4 | -6.15651 |
|  | XP\_065069482\_4468-model10.5 | -5.99256 |
|  | XP\_065069482\_4468-model10.6 | -5.99004 |
|  | XP\_065069482\_4468-model10.7 | -5.97488 |
|  | XP\_065069482\_4468-model10.8 | -5.94127 |
|  | XP\_065069482\_4468-model10.9 | -5.93881 |
|  | XP\_065069482\_4468-model10.10 | -5.91604 |
|  | XP\_065069482\_4468-model10.11 | -5.79326 |
|  | XP\_065069482\_4468-model10.12 | -5.54914 |
|  | XP\_065069482\_4468-model10.13 | -5.47262 |
|  | XP\_065069482\_4468-model10.14 | -5.04302 |
|  | XP\_065069482\_4468-model10.15 | -5.00286 |
|  | XP\_065069482\_4468-model10.16 | -4.86406 |
| # cluster 11 - Size: 9 |  |  |
|  | XP\_065069482\_4468-model11 | -6.51976 |
|  | XP\_065069482\_4468-model11.1 | -6.46507 |
|  | XP\_065069482\_4468-model11.2 | -6.22411 |
|  | XP\_065069482\_4468-model11.3 | -6.22252 |
|  | XP\_065069482\_4468-model11.4 | -6.08837 |
|  | XP\_065069482\_4468-model11.5 | -5.75031 |
|  | XP\_065069482\_4468-model11.6 | -5.65956 |
|  | XP\_065069482\_4468-model11.7 | -5.11429 |
|  | XP\_065069482\_4468-model11.8 | -4.73085 |
| # cluster 12 - Size: 2 |  |  |
|  | XP\_065069482\_4468-model12 | -5.90083 |
|  | XP\_065069482\_4468-model12.1 | -5.11086 |
| # cluster 13 - Size: 2 |  |  |
|  | XP\_065069482\_4468-model13 | -5.8868 |
|  | XP\_065069482\_4468-model13.1 | -5.34963 |
| # cluster 14 - Size: 3 |  |  |
|  | XP\_065069482\_4468-model14 | -5.54041 |
|  | XP\_065069482\_4468-model14.1 | -5.29067 |
|  | XP\_065069482\_4468-model14.2 | -4.63307 |
| # cluster 15 - Size: 1 |  |  |
|  | XP\_065069482\_4468-model15 | -5.49479 |
| # cluster 16 - Size: 3 |  |  |
|  | XP\_065069482\_4468-model16 | -5.37167 |
|  | XP\_065069482\_4468-model16.1 | -4.92941 |
|  | XP\_065069482\_4468-model16.2 | -4.64632 |
| # cluster 17 - Size: 1 |  |  |
|  | XP\_065069482\_4468-model17 | -5.19199 |
| # cluster 18 - Size: 1 |  |  |
|  | XP\_065069482\_4468-model18 | -5.07861 |
| # cluster 19 - Size: 2 |  |  |
|  | XP\_065069482\_4468-model19 | -4.91261 |
|  | XP\_065069482\_4468-model19.1 | -4.81405 |
|  |  |  |
